# Interepidemic Respiratory Syncytial Virus during the COVID-19 Pandemic

**DOI:** 10.1128/spectrum.00947-22

**Published:** 2022-04-25

**Authors:** ChunHong Huang, Malaya K. Sahoo, Michelle Verghese, Mamdouh Sibai, Daniel Solis, Kenji O. Mfuh, Jason Kurzer, Catherine A. Hogan, Thuy A. Doan, Benjamin A. Pinsky

**Affiliations:** a Stanford University School of Medicine, Stanford, California, USA; b Stanford Health Care, Stanford, California, USA; c Proctor Foundation, University of California San Francisco, San Francisco, California, USA; Johns Hopkins Hospital

**Keywords:** respiratory syncytial virus, pooling, whole-genome sequencing, metagenomic sequencing, phylogenetic analysis

## LETTER

Human respiratory syncytial virus (RSV) is an important cause of lower respiratory tract infection, resulting in significant morbidity and mortality in infants and young children, adults 65 years of age and older, and immunocompromised individuals (1). During the 2020 to 2021 respiratory virus season in the Northern Hemisphere, nonpharmaceutical interventions implemented to mitigate the COVID-19 pandemic, such as mask-wearing and social distancing, are thought to have disrupted the transmission of RSV (2).

In this context, we evaluated for interepidemic RSV in the San Francisco Bay Area by pooled screening of extracted eluates from 17,078 upper respiratory samples negative for SARS-CoV-2 and collected between 9 July and 30 November 2020 with an influenza A, influenza B, RSV multiplex, reverse-transcription quantitative PCR (FLUABR RT-qPCR). The pooled screening strategy, as well as the analytical and clinical validation of the FLUABR RT-qPCR, is described in detail in the supplemental material.

A single pool comprising 8 individual nucleic acid eluates was positive for RSV (*C_T_* [cycle threshold] value of 21.0). The original samples were reextracted, and RT-qPCR confirmed the presence of one RSV-positive sample (*C_T_* = 19.3). This represents a positivity rate of 0.006% (1/17,078). No influenza A- or influenza B-positive samples were identified. The RSV-positive specimen was collected mid-November 2020 from a 48-year-old woman who presented for drive-through SARS-CoV-2 respiratory specimen collection.

To characterize the genome of this interepidemic RSV, metagenomic RNA sequencing was performed as described previously (supplemental material) (3). A total of 32.5 million reads were acquired, 640 of which aligned to RSV-A and 39,576 of which aligned to RSV-B. Ten times coverage over 92.2% of the whole RSV-B genome was obtained, with mean coverage of 355×.

**FIG 1:**
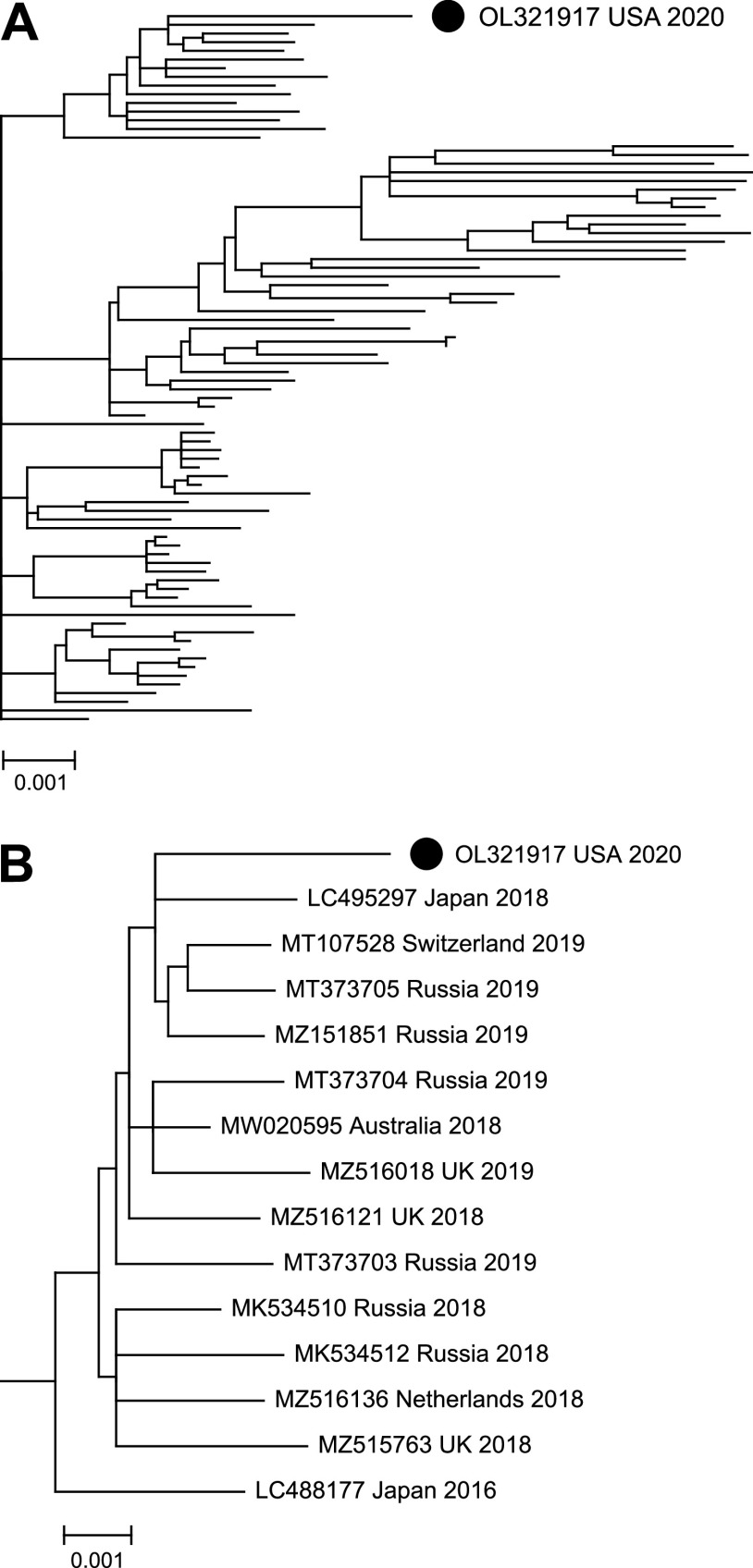
(A) Neighbor-joining phylogenetic tree representing 83 RSV-B whole-genome sequences from GenBank. Multiple sequence alignment was performed using Clustal X2, and the phylogenetic tree was rendered by MEGA X. (B) Close-up view of subtree showing closely related sequences with source country and year of specimen collection. Scale: 0.001 substitutions per nucleotide. The RSV-B whole-genome sequence identified in this study is identified by the filled-in black circle.

The consensus RSV-B whole-genome sequence (GenBank OL321917) was queried by BLAST against all 269 RSV-B complete whole-genome sequences deposited in GenBank from 2011 to 2021. After removal of duplicate and highly similar sequences (<0.2% difference), a neighbor-joining phylogenetic tree was generated (Fig. 1A). The most closely related whole-genome sequences were from a 2018 specimen collected in Japan (LC495297, 99.46% identity) and 2019 specimens collected in Russia (MT373705, 99.38% identity; MZ151851, 99.34% identity) and Switzerland (MT107528, 99.26% identity) (Fig. 1B).

There does not yet exist a unified genotype classification system for RSV. However, several approaches have been described, including genotyping based on the whole viral genome (Ramaekers [4] and Chen [5]), as well as the glycoprotein gene (G) ectodomain sequence (Goya [6]). The most closely related RSV-B whole-genome sequence common to all three classification systems was from a sample collected in England in 2013 (KY249660; 99.23% identity); the RSV-B in this study was genotype B6 (Ramaekers [4]), B.5.8 (Chen [5]), or B5.0.5a (Goya [6]).

In summary, we present a pooling strategy using existing extracted nucleic acids from specimens submitted for COVID-19 testing to identify interepidemic RSV-B, an accessible approach for ongoing evaluation of viral reservoirs. Metagenomic sequencing demonstrated a virus closely related to strains circulating in Asia and Europe prior to the COVID-19 pandemic, suggesting importation. Interepidemic sequences will contribute to a better understanding of seasonal RSV epidemics.
