# Supplementary material for: Interepidemic Respiratory Syncytial Virus during the COVID-19 Pandemic
Source: Microbiol Spectr. 2022 Apr 25;10(3):e00947-22. doi: 10.1128/spectrum.00947-22 (PMC9241888; doi:10.1128/spectrum.00947-22)

Supplemental Material

Ethics Statement. This study was reviewed and approved by the Institutional Review Board of Stanford University (protocol #48973).

Assay Design. The primer and probe sequences for the influenza A, influenza B, and respiratory syncytial virus (RSV) reverse transcription quantitative PCR (FLUABR RT-qPCR) are provided in Supplemental Table 1. The influenza A and B primers and probes were from the World Health Organization (WHO) protocol (https://www.who.int/influenza/gisrs_laboratory/molecular_diagnosis/en/) and Zhang *et al*. (1), respectively. The RSV primers and probe were based on those described in Kamau *et al.* (2), but redesigned to account for additional RSV-A and RSV-B sequences. The Kamau *et al.* nucleocapsid target region of sequence MG640062 was aligned against all RSV-A and RSV-B sequences as of June 2020 using the "somewhat similar sequences (blastn)" option in NCBI-BLAST with the "max target sequences" set to 5000. After removal of duplicate sequences, there were 91 unique RSV-A and 70 unique RSV-B sequences at least one base different from any other sequence in the set. The occurrences of A, T, G, and C at each position along the target were counted to locate regions with least variability and the primer sequences were adjusted to place the 3' ends on conserved bases. Two mismatches in the middle of the probe were converted to degenerate bases. Two RSV forward primers were included to accommodate sequence differences between RSV-A and RSV-B. RNaseP was used as internal control.

Nucleic acid extraction and RT-qPCR set-up. Total nucleic acids were extracted from 500 µL

on the QIA Symphony SP (Qiagen, Germantown, MD) using the QIAsymphony DSP Virus/Pathogen Midi Kit according to the manufacturer's recommendations, and eluted in 60 μL buffer AVE. RT-qPCR was performed using the Invitrogen Superscript III Platinum One-Step qRT-PCR kit (Invitrogen, Carlsbad, CA) on the Rotor-Gene Q instrument (Qiagen, Germantown, MD). Each 25 μL reaction contained 12.5 μL of 2X Buffer, 0.5 μL of enzyme mix, 2 μL of primer/probe mix, and 10 μL of eluate. Cycling conditions were as follows: hold at 52°C for 15 min, 94°C for 2 min, then 45 cycles of 94°C for 15 sec, 55°C for 40 sec, and 68°C for 20 sec. Fluorescence was analyzed in the crimson (influenza A), yellow (internal control: RNaseP), orange (RSV), and green (influenza B) channels; the threshold was set at 0.05 for the green and yellow channels, 0.07 for the orange channel, and 0.06 for the crimson channel. Any amplification curve crossing these thresholds was considered positive for the corresponding viral RNA. Samples were considered to have failed extraction or contain inhibitory substances if the internal control (IC) did not amplify at a cycle threshold ≤ 35 in samples negative for all three viruses.

Analytical Evaluation. 10-fold dilutions of quantified single-stranded DNA (ssDNA) (Supplemental Table 2) were run in triplicate revealing a linear range that extended from 8.08 to 2.08 $\log_{10}$ copies/mL for all three targets (Supplemental Figure 1).

The lower limit of 95% detection (LLOD) was determined using influenza A, influenza B and RSV positive specimens serially diluted using pooled negative nasopharyngeal swab samples in phosphate buffered saline. At least 95% of replicates were detected for each target at 50 copies/mL; 20/20 for influenza A, 19/20 for influenza B, and 19/20 for RSV.

Clinical Evaluation of the Multiplex RT-qPCR. A total of 128 archived clinical nasopharyngeal swab samples in viral transport media previously tested using Xpert Flu/RSV (Cepheid, Sunnyvale, CA) were selected to assess performance of the FLUABR RT-qPCR. These samples included 33 positive influenza A, 31 positive influenza B, 32 positive RSV, and 32 samples negative for all three targets. Positive percent agreement was 100% (33/33) for influenza A, 96.8% (30/31) for influenza B, and 100% (32/32) for RSV. The single discrepant influenza B specimen was tested using the Panther Fusion FluA/B/RSV Assay (Hologic, Marlborough, MA) and found to be negative. Negative percent agreement was 98.9% (94/95) for influenza A, 100% (97/97) for influenza B, and 95.9% (92/96) for RSV. Three of the four RSV samples that were negative by Xpert Flu/RSV but positive by FLUABR RT-qPCR were positive when tested using the Panther Fusion FluA/B/RSV Assay. The single influenza A sample negative by Xpert Flu/RSV but positive by FLUABR RT-qPCR was negative when tested using the Panther Fusion FluA/B/RSV Assay.

Screening of Clinical Samples submitted for SARS-CoV-2 RT-qPCR. Total nucleic acids were extracted from 500 µL on the QIA Symphony SP (Qiagen, Germantown, MD) using the QIAsymphony DSP Virus/Pathogen Midi Kit according to the manufacturer's recommendations, and eluted in 60 µL buffer AVE. These included 11,183 individual samples, as well as pools of 2 (n=13), 3 (n=51), and 4 (n=1429) samples negative for SARS-CoV-2 RNA. Seventeen individual eluates were tested using the FLUABR RT-qPCR. The remaining eluates were combined in pools of 2 to 9 eluates. A total of 17,078 upper respiratory samples were screened using the FLUABR RT-qPCR.

RSV Sequencing and Analysis. Metagenomic RNA sequencing was performed as described previously (3). Briefly, 5 μL of extracted total nucleic acids was converted to cDNA and sequencing libraries were prepared using the NEBNext RNA ULTRA II kit (New England Biolabs) according to the manufacturer's instructions. Samples were sequenced on the NovaSeq 6000 system (Illumina) using 150-nucleotide paired-end sequencing. Sequences were initially aligned to reference RSV-A (GenBank: MG642067.1) and RSV-B (MG642053.1) whole genome sequences using the bwasw module in BWA (version 0.7.9a-r786) with match score 2 and mismatch penalty -3 (options -a2 -b3).

Consensus whole genome sequence was generated from the RSV-B alignment bam file using samtools (version 0.1.19) mpileup followed by bcftools vcf2fq using default parameters. This consensus sequence was used to query GenBank and the resulting top BLAST hit was an RSV-B sequence (LC495297) from a specimen collected in Japan in August 2018 from a pediatric patient with respiratory failure. Using LC495297 as reference, the alignment and consensus generation was repeated as above, and the new consensus sequence was deposited in GenBank (OL321917).

Supplemental Figure Legend

Supplemental Figure 1. Linearity of the influenza A, influenza B, respiratory syncytial virus (RSV) reverse transcription quantitative PCR using serial 10-fold dilutions of single-stranded DNA run in triplicate from 8.08 to 2.08 log_{10} copies/mL. Influenza A (A), influenza B (B), and RSV(C). The solid lines represent the linear least squares regression.

Supplemental References

1. Zhang H, Wang Y, Porter E, Lu N, Li Y, Yuan F, Lohman M, Noll L, Zheng W, Stoy C, Lang Y, Huber VC, Ma W, Peddireddi L, Fang Y, Shi J, Anderson G, Liu X, Bai J. 2019. Development of a multiplex real-time RT-PCR assay for simultaneous detection and differentiation of influenza A, B, C, and D viruses. Diagn Microbiol Infect Dis 95:59-66.
2. Kamau E, Agoti CN, Lewa CS, Oketch J, Owor BE, Otieno GP, Bett A, Cane PA, Nokes DJ. 2017. Recent sequence variation in probe binding site affected detection of respiratory syncytial virus group B by real-time RT-PCR. J Clin Virol 88:21-25.
3. Doan T, Sahoo MK, Ruder K, Huang C, Zhong L, Chen C, Hinterwirth A, Lin C, Gonzales JA, Pinsky BA, Acharya NR. 2021. Comprehensive pathogen detection for ocular infections. J Clin Virol 136:104759.

Supplemental Table 1: Influenza A, Influenza B, and RSV Primer and Probe Sequences

Name	Sequence (5'to 3')	Final Concentration (nM)
**Primers**		
InfA-F	GACCRATCCTGTCACCTCTGAC	300
InfA-R	AGGGCATTYTGGACAAAKCGTCTA	300
InfBv1-Fa	GCAGAGCAGCGAGATCTTCAG	300
InfBv1-R	CTTTYCCCATTCCATTCATTGT	300
RSV_N1_Fa	GAAGATGCAAATCATAAATTCACAGG	300
RSV_N1_Fb	GAGGATGCTAACAACAAACTTACTGG	300
RSV_N1_R	TGATATCCMGCATCTTTRAGTAT	300
RNAseP_Fwd	AGATTTGGACCTGCGAGCG	100
RNAseP_Rev	GAGCGGCTGTCTCCACAAGT	100
**Probes**		
InfA-prb_Q705	TGCAGTCCTCGCTCACTGGGCACG	100
InfBv1-prb_FAM	CTGTGTTCATAGCTGAGACCATCTGC	100
RSV_N1_prb_cf610	ATGTCYAGRTTAGGAAGRGAAG	100
RNAseP Probe cf560	TTCTGACCTGAAGGCTCTGCGCG	50

InfA, influenza A; InfB, influenza B; RSV, respiratory syncytial virus; Q705, Quasar 705; FAM, 6-carboxyfluorescein; cf610, CalFluor 610; cf560, CalFluor 560

Primers were purchased from Integrated DNA Technologies (San Diego, California) and hydrolysis probes were purchased from Biosearch Technologies (Petaluma, California).

Table 2. Influenza A, Influenza B, and RSV single-stranded DNA Sequences

Virus	Sequence	GenBank Reference
FluA-ssDNA	CTAAAGACAAGACCAATCCTGTCACCTCTGACTA<br>AGGGGATTTTAGGATTTGTGTTCACGCTCACCGTG<br>CCCAGTGAGCGAGGACTGCAGCGTAGACGCTTTG<br>TCCAAAATGCCCTTAATGGGAAT	MT624438.1<br>matrix protein 2 (M2)
FluB-ssDNA	TCCTGGAAATTATTCAATGCAAGTAAAACTAGGA<br>ACGCTCTGTGCTTTATGCGAGAAACAAGCATCAC<br>ATTCACACAGGGCTCATAGCAGAGCAGCGAGATC<br>TTCAGTGCCTGGAGTGAGACGAGAAATGCAGATG<br>GTCTCAGCTATGAACACAGCAAAAACAATGAATG<br>GAATGGGAAAAG	MT600428.1<br>matrix protein 1 (M1)
RSV-N1-ssDNA	ATTAATCACTGAAGATGCAAATCATAAATTCACA<br>GGATTAATAGGTATGTTATATGCTATGTCCAGGTT<br>AGGAAGGGAAGACACTATAAAGATACTTAAAGA<br>TGCTGGATATCATGTTAAAGCT	MG642062.1<br>Nucleoprotein (N)

FluA, influenza A; FluB, influenza B; RSV, respiratory syncytial virus; ssDNA, single-stranded DNA
Purchased from Integrated DNA Technologies (San Diego, California)

Supplemental Figure 1

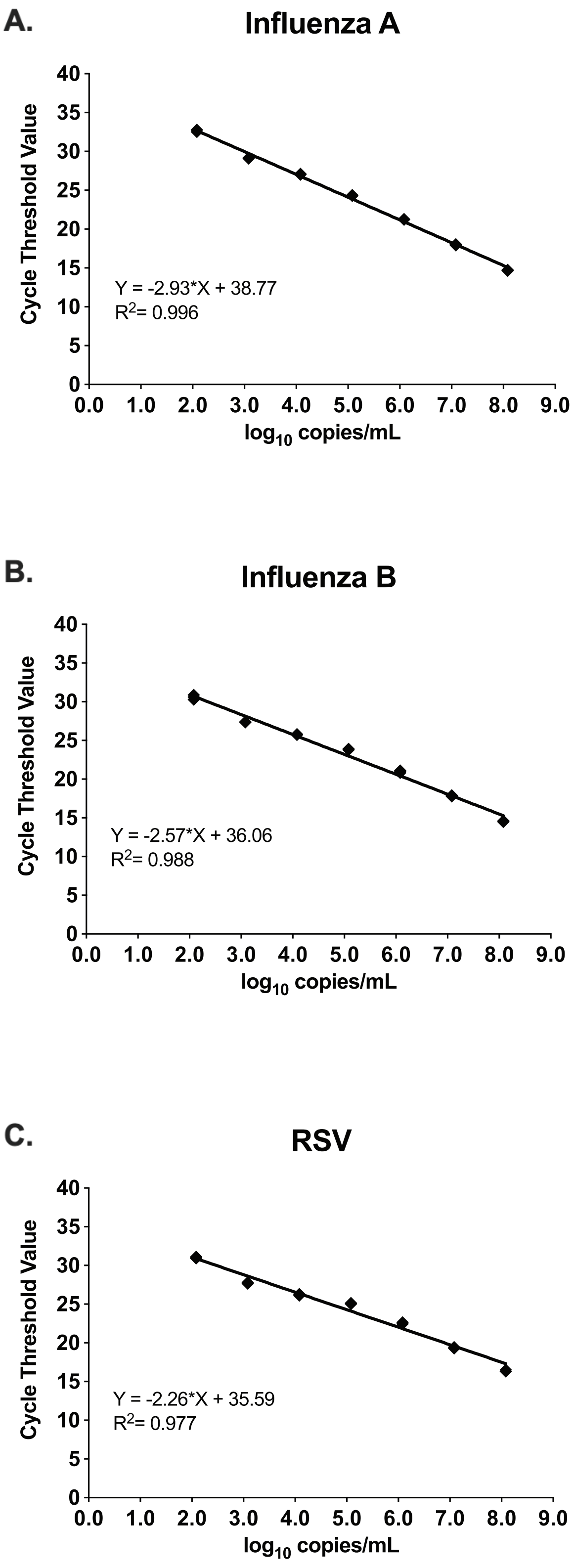

Supplement: SUPPLEMENTAL FILE 1 — Supplemental material. Download spectrum.00947-22-s001.pdf, PDF file, 0.3 MB [file spectrum.00947-22-s001.pdf]
